# Original Nerve Growth Factor Mimetic Dipeptide GK-2 Restores Impaired Cognitive Functions in Rat Models of Alzheimer’s Disease

**Published:** 2013

**Authors:** P.Yu. Povarnina, O.N. Vorontsova, T.A. Gudasheva, R.U. Ostrovskaya, S.B. Seredenin

**Affiliations:** Zakusov Institute of Pharmacology, RAMS, Baltiyskaya Str., 8, Moscow, Russia, 125315

**Keywords:** low molecular mimetic of NGF, GK-2, septo-hippocampal transsection, streptozotocin model of Alzheimer’s disease, habituation, Morris water maze

## Abstract

Dipeptide mimetic of the nerve growth factor (NGF) loop 4, hexamethylenediamide bis-(N-monosuccinyl- glutamyl-lysine) (GK-2), was synthesized at the V.V. Zakusov Scientific Research Institute of Pharmacology of the Russian Academy of Medical Sciences. GK-2 exhibited in vitro neuroprotective activity at nanomolar concentrations, was efficient in animal models of the Parkinson’s disease, ischemic and hemorrhagic stroke, and global cerebral ischemia at doses of 0.01–5 mg/kg (intraperitoneally) and 10 mg/kg (per os). The mnemotropic effects of subchronic intraperitoneal administration of GK-2 on rat models of the Alzheimer’s disease are described in this paper. Dipeptide GK-2 at a dose of 1 mg/kg is found to decrease the habituation deficit induced by the septo-hippocampal pathway transsection and, at a dose of 0.5 mg/kg, to significantly prevent spatial memory impairment in Morris water maze induced by intracerebral injection of streptozotocin. Thus, GK-2, an original dipeptide mimetic of NGF, acts on models of the Alzheimer’s disease upon systemic administration.

## INTRODUCTION

The Alzheimer’s disease (AD) is the most common cause of dementia. The number of AD patients is expected to double by 2050 [1]. No pharmacological agents capable of providing long-term neuroprotection to AD patients or limiting the development of cognitive impairment area vailable at the moment [2].

The contribution of the nerve growth factor (NGF) to the pathogenesis of AD has been well documented. The progressive decline in cognitive functions in AD patients is associated with the degeneration of cholinergic neurons in the basal forebrain [3], which are the primary target of this neurotrophin in the central nervous system. This neurotrophin ensures the preservation of the biochemical and morphological phenotypes of the aforementioned neurons and their survival in the presence of damaging factors [4]. NGF inhibits the formation of amyloid plaques and neurofibrillary tangles in the brain – the main pathomorphological characteristics of AD – via the inhibition of amyloidogenic processing of APP [5] and hyperphosphorylation of the tau protein, which is involved in the formation of neurofibrillary tangles [6].

The exact reasons behind sporadic AD still remain unknown; however, evidence is accumulating in support of the hypothesis of the deterioration of trophic maintenance of the cholinergic neurons of the basal forebrain by NGF as a trigger of the disease [7, 8]. It was demonstrated in AD11 transgenic mice (anti-NGF antibodies develop in these mice during the postnatal period) that chronic NGF deprivation causes a cholinergic deficit, loss of neurons and synapses, formation of amyloid plaques and neurofibrillary tangles, a decrease in synaptic plasticity, and memory deficit [8]. Intranasal administration of NGF to AD11 mice prevented the disruption of cholinergic transmission, the accumulation of β-amyloid and the hyperphosphorylated tau protein, and the development of memory deficit [9].

A variety of experimental AD models (such as the destruction of the basal nuclei of Meynert by ibotenic acid, septo-hippocampal transsection and natural aging) have shown that therapeutic intracerebral NGF administration counteracts the degeneration of cholinergic neurons and restores cognitive functions [10-12].

Poor pharmacokinetic properties and the limited ability to penetrate through the blood–brain barrier and pleiotropy limit the medical application of native NGF. Hence, a number of pharmaceutical companies and research groups are currently in the process of developing low-molecule-weight NGF mimetics [13-15].

Dimeric dipeptide mimetic GK-2 (hexamethylenediamide bis-(N-monosuccinyl-glutamyl-lysine)) was synthesized based on the structure of the β-turn of the NGF loop 4 at the V.V. Zakusov Scientific Research Institute of Pharmacology of the Russian Academy of Medical Sciences [16]. Identically to NGF, GK-2 causes the phosphorylation of specific TrkA receptors and the kinases Akt that participate in the manifestation of the neuroprotective effects mediated by these receptors [17]. GK-2 at nanomolar concentrations exhibited a high NGF-like neuroprotective activity during *in vitro* experiments. However, in contrast to the native protein, GK-2 demonstrated no signs of differentiating activity [18]. GK-2 prevented H_2_O_2_- or glutamic-acid-induced destruction of immortalized mouse hippocampal neurons in culture (HT-22 line) and protected rat pheochromocytoma PC12 cells from the action of neurotoxin MPTP (1-methyl-4-phenyl-1,2,3,6-tetrahydropyridine) [19]. Dipeptide GK-2 exhibited neuroprotective properties and improved cognitive functions [20-22], and reduced the severity of behavioral symptoms in a number of models of the parkinsonian syndrome [23] in experimental models of acute and chronic cerebral ischemia in rats. Dipeptide GK-2 exhibits low toxicity (LD_50_ = 714 mg/kg upon intravenous administration to male mongrel mice) and shows none of the major side effects typical of NGF. It does not reduce the pain threshold (at doses of 0.5–2 mg/kg i/p in a tail flick test after thermal stimulation, water temperature of 55°C) [24] and does not cause weight loss upon chronic administration to rats (0.5 mg/kg, i/p) [25].

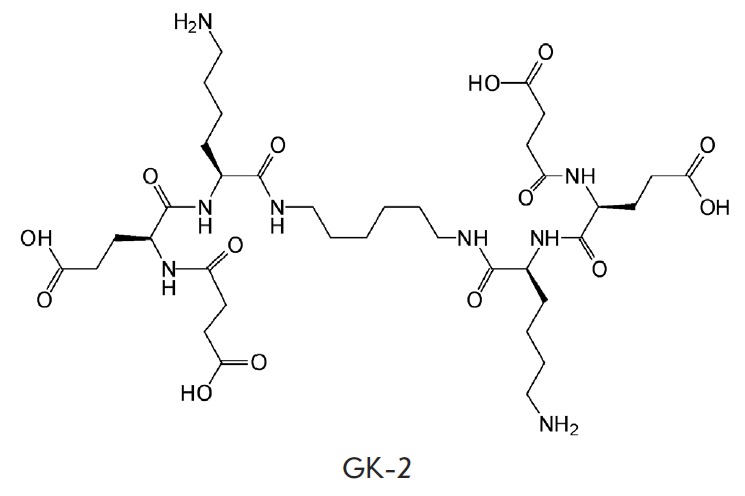



The aim of this study was to investigate the mnemotrophic effects of GK-2 on AD models. There are two main approaches to the modeling of the abnormalities typical of AD: surgical and neurotoxic. The former approach entails the transsection of the septo-hippocampal pathway, which is a well-researched and widely used AD model. Deafferentation of the hippocampus attributed to septo-hippocampal transsection results in the development of cholinergic deficiency and the related disorder of cognitive functions [26, 27]. This model has been used by various researchers to study the effects of NGF [28, 29]; hence our decision to use it to evaluate the activity of the NGF mimetic GK-2. A streptozotocin AD model reproducing the major pathological processes in the brain (including the accumulation of β-amyloid and the hyperphosphorylated tau protein, as well as the spatial memory abnormalities [30] that are also typical of AD patients) was used as a neurotoxic model [31].

## EXPERIMENTAL

**Substances**

Dipeptide GK-2 (hexamethylenediamide bis-(Nmonosuccinyl- glutamyl-lysine), molecular weight of 835 Da) was synthesized at the V.V. Zakusov Scientific Research Institute of Pharmacology of the Russian Academy of Medical Sciences [16]. Nembutal (Sigma, USA) was used for anesthesia. Memantine (1-amino- 3,5-dimethyladamantane hydrochloride, molecular weight of 215 Da) was purchased from the Merck KgaA company (Germany).

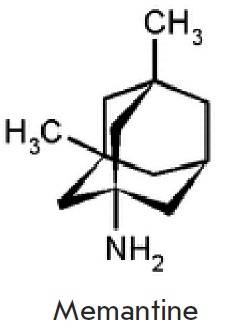



Nembutal was administered in the form of a saline suspension, dipeptide GK-2 was dissolved in distilled water, and memantine was administered in the form of a distilled water suspension. All substances (except streptozotocin) were administered i/p at a dose of 2 ml/ kg of body weight.

The GK-2 doses (0.5 and 1 mg/kg) were selected as the ones most efficient *in vivo *according to the data obtained in a study of the dose-dependent effect of this compound in the haloperidol catalepsy model in rats used for screening compounds with potential antiparkinsonian activity [23]. The efficacy of these doses was also confirmed in various cerebral ischemia models [20-22].

**Animals**

The experiments were conducted on 32 male mongrel white rats and 24 male Wistar rats obtained from the animal nursery “Stolbovaya” of the Russian Academy of Medical Sciences. The animals were kept in a vivarium under *ad libitum *feeding and natural light conditions. Behavioral experiments were performed during winter between 10.00 and 14.00 hours, local time. The requirements regarding the use of animals for experimental research specified in the European Council Directives 86/609/EE S were abided by during the experiments on the rats.

**Investigations into the effects of GK-2 on an AD model with septo-hippocampal pathway transsection [26]**

*Study design*. The Wistar rats (280–400 g) were randomly divided into three groups: rats subjected to sham surgery (*n*=6); rats subjected to surgery (*n*=10); and rats that were subjected to surgery and received dipeptide GK-2 (*n*=8). Dipeptide GK-2 (1 mg/kg) was administered 2 h after the surgery and subsequently every 48 h (a total of seven injections). The “sham surgery” and “surgery” groups received distilled water instead of GK-2, according to the same scheme and in equivalent volumes. The animals were subjected to an open-field test for 48 h after the last injection of GK-2 (or distilled water).

*Surgery*. A Nembutal-anesthetized (60 mg/kg) and scalped animal was mounted onto a stereotaxic apparatus. Transverse incisions 1 mm wide were made in the cranial bone: the starting point of the incision localized at the level of bregma (AP = 0.0) and 2 mm laterally relative to the latter (L = ± 2.0); the endpoint of the incision localized 2 mm caudally relative to the bregma and 1 mm laterally relative to the latter (AP = –2.0; L = ± 1.0). After the bone had been cut, the dura matter was incised in the area of incisions. A sterile bent needle was placed into the incision to a depth of 6.2 mm from the bone surface (DV = +6.2). Simultaneous manipulation using two stereotaxic screws allowed to relocate the needle almost to the endpoint of the transverse incision. The needle was then slowly lifted from the incision. The procedure was repeated twice on each hemisphere. The sham surgery procedure was similar to that of transsection with one exception: the needle was placed to a depth of 3 mm from the bone surface (DV = +3.0).

*Open-field test*. This test is commonly used to assess general motor and exploratory activities [32]. The setup consisted of a circular arena made of white PVC. The arena diameter was 90 cm; the walls were 40 cm high. The floor of the arena was divided into 19 squares of approximately equal area with lines. The animal was carefully placed on the floor in the center of the arena; the number of squares and vertical stands crossed by it during 4 min was recorded using the RealTimer software program (Scientific and Production Company “Open science,” Russia). Transsection of the septo-hippocampal pathway in rats is known to cause impairment of the extinction of the exploratory orientation reaction (EOR) in the open field test [33, 34]. The EOR extinction coefficient, which represents the ability of rats to exhibit habituation, was calculated according to the equation *C_e_ = a/b*, where *a *is the number of squares crossed in the group during the first minute of observation, and *b *is the number of squares crossed in the group during the last minute of observation.

**Investigations into the effects of GK-2 on the AD model associated with the administration of streptozotocin into cerebral ventricles [30]**

*Study design. *The mongrel male rats (330–380 g) were randomly allocated into four groups: those subjected to sham surgery (n = 6); those subjected to surgery (n = 9), those subjected to surgery and treated with dipeptide GK-2 (n = 7), and those subjected to surgery and treated with the reference drug, memantine (n = 6). Dipeptide GK-2 (0.5 mg/kg or 6 X 10^-7^ mol/kg) was administered 4 h after the surgery and then once daily for 2 weeks (a total of 14 injections). Memantine was administered according to the same scheme at a dose of 10 mg/kg (4.6 X 10^-5^ mol/kg). This dose was selected as the most effective one according to the published data [35]. Distilled water was administered to the “sham surgery” and “surgery” groups instead of GK-2 or memantine according to the same scheme in equivalent volumes. Three weeks after the surgery, the rats were trained to search for a submerged platform in the “Morris water maze” setup for 5 days. One week after the completion of the training, the rats were tested for retention of this skill.

*Surgery. *A Nembutal-anesthetized rat (60 mg/kg) was mounted onto a stereotactic apparatus. Streptozotocin in a Ringer’s solution at a dose of 3 mg/kg was bilaterally injected into the cerebral ventricles of the animal (5 μl per ventricle) according to the coordinates AP = –1; L = ±1.5; DV = +3.5. A total of 5 μl of the Ringer’s solution was injected into the cerebral ventricles of the sham-operated animals. The injections were carried out at a rate of 1 μl/min with a pause after each microliter (1 min). Upon completion of the administration, the needle was left in place for an additional 3 min and then removed.

Morris water maze. This test, first proposed by Morris D. [36], is mainly used to assess spatial memory. Our experimental setup consisted of a pool made of gray plastic (150 cm in diameter, 60 cm high). The pool was filled with water (24–25°C) to a depth of 40 cm. The pool was conventionally divided into four sections. A platform measuring 10 cm in diameter and submerged in water to a depth of 2 cm was located in the center of one of the sections. Various visual stimuli were placed on the walls of the room facing each of the conventional sectors. The rats were taught to find the submerged platform for 5 days. During this period, the animals were placed in water at 4 different starting positions near the pool wall (starting positions were located in the centers of the conventional sectors) four times per day. The sequence of starting positions was identical for all animals during the day and was changed every day. The interval between the placements was 30 s. After a rat reached the platform, it was left on it for 5 s. If an animal could not find the submerged platform within 60 s, it was gently guided to it. After 1 week, the test for retention of the acquired skill of finding the platform was carried out. During the test the animals were again placed in water 4 times at different starting positions. Escape latency (EL) was recorded for each attempt.

*Statistical analysis. *Statistical processing was carried out using the Statistica 10.0 software. Reduction of the average daily EL during the training period was used as a criterion for spatial learning ability in the Morris water maze setup. The average EL on the day of the test, as well as the EL during the fourth and last test placement, was used as a criterion of safety and effectiveness of reproduction of the acquired skill. In order to carry out the analysis of intergroup differences in the Morris water maze setup, a univariate analysis of variance (one-way ANOVA) and a Fisher’s multiple comparison test were utilized. The Wilcoxon test was used for pairwise comparison of the related samples. The data are presented as medians of the samples, lower and upper quartiles. The results were considered statistically significant at p < 0.05.

The therapeutic effect (T_e_) of dipeptide GK-2 was calculated using the equation:

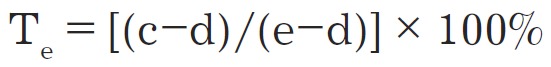



where c is the value of the parameter in the “surgery + treatment” group, d is the value of the parameter in the “surgery” group, and e is the value of the parameter in the “sham surgery” group.

## RESULTS AND DISCUSSION

**Dipeptide GK-2 counteracts the septohippocampal pathway transsection-induced impairment of cognitive functions in rats**

No statistically significant intergroup differences in the intensity of horizontal and vertical motor activities were identified. The analysis of the dynamics of the horizontal locomotor activity (number of squares crossed) demonstrated that this parameter in shamoperated animals decreased significantly and 4 minutes into the test was significantly lower than that during the 1^st^ minute (C_e_ = 2.3). Rodents are characterized by gradual extinction of EOR upon encountering a new situation, which is attributed to addiction or habituation. Transsection of the septo-hippocampal pathway led to impairment of EOR extinction over time, which was reflected at an almost constant level of the horizontal locomotor activity in the rats subjected to surgery over the entire period of testing (C_e_ = 1). Similar results were also obtained by other researchers using this model [33, 34]. Impairment of the extinction of EOR under new conditions caused by the septo-hippocampal transsection was probably associated with abnormalities of the spatial memory [33]. The animals subjected to surgery and treated with GK-2 demonstrated recovery of the ability to habituate (C_e_ = 1.9) (Table 1). The therapeutic effect of GK-2 was approximately 70%.

**Table 1:** Effect of GK-2 on the impairment of EOR extinction during the open-field test caused by transsection of the septo-hippocampal pathway

Group	min 1	min 2	min 3	min 4	Extinction coefficient
Sham surgery	21(15–32)	14(11–25)	9.5(7–16)*	9(8–13)*	2.3
Surgery	11.5(8–35)	12(9–32)	19(11–24)	11.5(9–29)	1
Surgery + GK-2	12.5(9–21)	11.5(7–14)	15.5(8–17)	6.5(3–8)*	1.9

* p < 0.05 as compared to the locomotor activity in the same group during the first minute of the test.* Note. *The data are presented as medians of respective samples. The extinction coefficient (*C_e_*) of the EOR reflecting the ability of rats to habituate was calculated using the equation *C_e_*= *a/b*, where *a *is the number of squares crossed in the group during the first minute of observation, and *b *is the number of squares crossed in the group during the last minute of observation.

**Dipeptide GK-2 completely restores the abnormality of spatial memory caused by the administration of streptozotocin into the cerebral ventricles of rats**

Intracerebral administration of streptozotocin is known to cause the development of cognitive deficit, which can be detected within 2 weeks after surgery and progresses for several months [30]. In particular, intracerebral administration of streptozotocin causes abnormalities in spatial memory during the Morris water maze test. This disorder correlates with biochemical changes in the hippocampus (decreased activity of choline acetyltransferase [30]), as well as with a significant decrease in the immunoreactivity of the transcription factor CRE B, which plays an important role in the regulation of learning and the memory processes participating in the transformation of short-term memory into longterm memory [37].

**Fig. 1:**
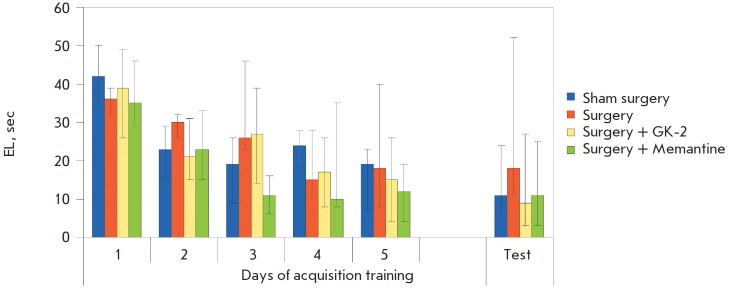
Spatial learning and skill retention test in the Morris water maze. Sham surgery – bilateral administration of a Ringer’s solution into the cerebral ventricles of rats plus subchronic intraperitoneal administration of distilled water. Surgery – bilateral administration of streptozotocin dissolved in the Ringer’s solution into the cerebral ventricles of rats plus subchronic intraperitoneal administration of distilled water. Surgery + GK-2 – bilateral administration of streptozotocin dissolved in the Ringer’s solution into the cerebral ventricles of rats plus subchronic intraperitoneal administration of dipeptide GK-2. Surgery + memantine – bilateral administration of streptozotocin dissolved in the Ringer’s solution into the cerebral ventricles of rats plus subchronic intraperitoneal administration of memantine. The EL per four placements within 1 day were averaged for each animal. The data are presented as medians and interquartile ranges

In this study, the EL decreased during the learning period in all groups; no statistically significant intragroup differences were identified during this period ( Fig. 1 ). Hence, administration of streptozotocin had no effect on the learning ability of animals during the Morris water maze test. This can be attributed to the fact that the training was either conducted 3 weeks after the surgery (the cognitive deficit was probably not sufficiently pronounced during this period) or that mongrel rats were used. Intracerebral administration of streptozotocin in the study conducted by Shingo et al. [37] also had no effect on the ability of Wistar rats to learn in the Morris water maze test 2 weeks after the surgery.

**Fig. 2:**
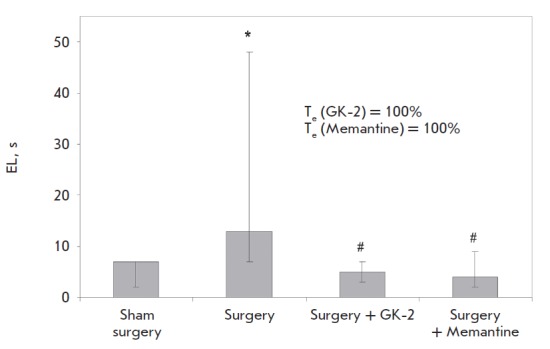
Effects of GK-2 on the retention of the skill of finding the submerged platform in the Morris water maze setup, which is deficient in rats with experimental AD induced by the injection of streptozotocin into the cerebral ventricles. The data are presented as medians and interquartile ranges. * – *p *= 0.017 in comparison to the “sham surgery” group; # – p = 0.012 (“surgery + GK-2”) and 0.014 (“surgery + memantine”) in comparison with the “surgery” group (oneway ANOVA with subsequent post hoc Fisher test). The therapeutic effect (*T_e_*) of dipeptide GK-2 was calculated using the following equation: *T_e_*= [(*c–d*)/(*e–d*)] X 100%, where *c *is the EL in the “surgery + treatment” group, *d *is the EL in the “surgery” group, and *e *is the EL in the “sham surgery” group

The test for the retention of the acquired skill carried out 1 week after the learning phase had been completed demonstrated that the average (over four placements) EL in the sham-operated rats was characterized by a tendency to decrease as compared to the last day of learning, which is consistent with the data on the increased level of reproduction of long-term spatial memory during later periods after the learning phase [38]. Meanwhile, the EL of the rats subjected to surgery did not decrease as compared to the last day of learning and was higher than that in the “sham surgery” group, although these results were not statistically significant. The EL among rats receiving GK-2 did not differ from that for the “sham surgery” group ( Fig. 1 ). The analysis of intergroup differences with respect to EL during the last (fourth) placement in the test showed that this parameter in rats subjected to surgery was significantly higher than that in the “sham surgery” group (Fig. 2, Table 2). NGF mimetic GK-2 fully prevented this abnormality and was equal in terms of its effectiveness to the reference drug memantine (Fig. 2).

**Table 2:** Effects of GK-2 on EL in the Morris water maze in rats with experimental AD induced by administration of streptozotocin into the cerebral ventricles

Group	EL*
Sham surgery	7 (3–7)
Surgery	13 (7–48)
Surgery + GK-2	5 (4–6.5)
Surgery + Memantine	4 (2–8)

* Data are presented as medians; interquartile ranges are given in parentheses.

Hence, dipeptide GK-2 can counteract the cognitive deficit in rat AD models.

It has previously been established that GK-2 has neuroprotective activity and acts via an NGF-like mechanism [18]. Intracerebral administration of NGF is known to restore cognitive functions in *in vivo *AD models. Thus, administration of mouse NGF into the cerebral ventricles of rats subjected to septo-hippocampal transsection over a period of 14 days significantly improved the spatial memory in the Morris water maze test with a therapeutic effect of approximately 75% [28]. The recovery of cholinergic neurons under the influence of exogenous NGF 1 month after fimbria-fornix transsection in rats was described in [29]. Improvement of the spatial memory in rats treated with NGF 2 weeks after the surgery can be associated with increased survival of cholinergic neurons and/or improvement of cholinergic transmission in the hippocampus [28]. Intracerebral administration of recombinant human NGF to rats for 3 weeks after the destruction of the basal nuclei of Meynert by ibotenic acid partially prevented the impairment of the learning ability in the Morris water maze with a therapeutic effect of approximately 25% [39]. Administration of recombinant human NGF resulted in a decrease in the body weight of rats. In addition to weight loss, such side effects of NGF as pain syndrome [40, 41], Schwann cell hyperplasia, and multiple sprouting of sympathetic and sensory axons in the medulla and the spinal cord have been observed [42].

Low molecular weight mimetics of NGF exhibiting activity in AD models have been described. A nonpeptide mimetic of NGF, the selective agonist of TrkAreceptors known as D3, restored cholinergic deficit and improved spatial memory in aged animals in the Morris water maze upon intracerebral administration (40 μg per rat) [43].

Another low-molecular-weight non-peptide mimetic of NGF known as MT2, which is also a TrkA-receptor agonist, exhibits neuroprotective and antiamyloidogenic activity in cellular AD models at a concentration of 5–30 μmol/ml [44].

No data regarding the systematic application of both NGF and its low-molecular-weight mimetics activating TrkA-receptors in AD models have been obtained. Only a non-peptide agonist of p-75 receptors known as LM11A-31, which demonstrated neuroprotective and antiamnestic activities in a genetic mouse model of AD, has been identified [45].

## CONCLUSIONS

Hence, data on the mnemotropic effects of the dipeptide mimetic of NGF causing the phosphorylation of TrkA-receptors and Akt-kinases upon systemic administration in AD models have been obtained for the first time.

Dipeptide GK-2 considerably prevents the abnormalities of habituation caused by septo-hippocampal transsection. Identically to memantine, which is widely used for treatment of AD, GK-2 significantly prevents spatial memory deficit in the Morris water maze in the streptozotocin AD model. The effective dose of GK-2 by weight is 20 times lower than that of memantine; the effective dose by amount of matter is 80 times lower.

The results obtained suggest that further development of GK-2 as a neuroprotective medicinal product that could prevent the development of AD is rather promising.

## Abbreviations

AD: Alzheimer’s disease
NGF: nerve growth factor
APP: amyloid precursor protein
i/p: intraperitoneally
EL: escape latency
EOR: exploratory orientation reaction
